# “Teedrogen und Phytopharmaka”

**DOI:** 10.3797/scipharm.br-11-02

**Published:** 2011-09-30

**Authors:** Wolfgang Kubelka

**Affiliations:** Department of Pharmacognosy, University of Vienna, Austria

The last German edition of this book was published in 2002, the last and 3rd English edition: “Herbal Drugs and Phytopharmaceuticals”, was printed not later than 2004, and no newer editions were available. Therefore everybody interested in Herbal Drugs and Herbal Medicinal Products, including non German speaking readers, appreciates very much this new publication. During the last years, medicinal and pharmaceutical research in the field of herbals has grown enormously, especially concerning active constituents, analytics, and application in therapy. This is shown by the evaluation and quotation for the book of more than 700 papers published between 2002 and 2008, and the addition of 22 new monographs of medicinal plants. Altogether 234 herbal drugs are described with informative pictures of the plants and their respective drugs and structural formulae of their constituents. Where important for identification, microscopical characters, hand drawings and TLCs are given. For each monograph the chapter “Indications” has been restructured and made even more suitable and useful for daily practice.

The aim of the editor, to present a “handbook for practice on a scientific basis”, has been fully achieved. The amendments of this new edition make it undispensible for the owners of the former ones, be it the last English or the German version.

I am sure that everybody working with medicinal plants or using herbs and herbal products for therapy, will like to have the newest edition of this real classic on his/her desk for rapid and serious information.

